# Gene delivery corrects N-acetylglutamate synthase deficiency and enables insights in the physiological impact of L-arginine activation of N-acetylglutamate synthase

**DOI:** 10.1038/s41598-021-82994-8

**Published:** 2021-02-11

**Authors:** P. Sonaimuthu, E. Senkevitch, N. Haskins, P. Uapinyoying, M. McNutt, H. Morizono, M. Tuchman, L. Caldovic

**Affiliations:** 1 Center for Genetic Medicine Research, Children’s Research Institute, Children’s National Medical Center, 111 Michigan Ave NW, Washington, DC 20010U USA; 2 National Institute of Neurological Disorders and Stroke, National Institutes of Health, Bethesda, MD USA; 3 Children’s Medical Center, UT Southwestern Medical Center, Dallas, TX USA

**Keywords:** Biochemistry, Molecular biology, Molecular medicine

## Abstract

The urea cycle protects the central nervous system from ammonia toxicity by converting ammonia to urea. N-acetylglutamate synthase (NAGS) catalyzes formation of N-acetylglutamate, an essential allosteric activator of carbamylphosphate synthetase 1. Enzymatic activity of mammalian NAGS doubles in the presence of L-arginine, but the physiological significance of NAGS activation by L-arginine has been unknown. The NAGS knockout (*Nags*^−/−^) mouse is an animal model of inducible hyperammonemia, which develops hyperammonemia without N-carbamylglutamate and L-citrulline supplementation (NCG + Cit). We used adeno associated virus (AAV) based gene transfer to correct NAGS deficiency in the *Nags*^*−/−*^ mice, established the dose of the vector needed to rescue *Nags*^*−/−*^ mice from hyperammonemia and measured expression levels of *Nags* mRNA and NAGS protein in the livers of rescued animals. This methodology was used to investigate the effect of L-arginine on ureagenesis in vivo by treating *Nags*^*−/−*^ mice with AAV vectors encoding either wild-type or E354A mutant mouse NAGS (mNAGS), which is not activated by L-arginine. The *Nags*^−/−^ mice expressing E354A mNAGS were viable but had elevated plasma ammonia concentration despite similar levels of the E354A and wild-type mNAGS proteins. The corresponding mutation in human NAGS (NP_694551.1:p.E360D) that abolishes binding and activation by L-arginine was identified in a patient with NAGS deficiency. Our results show that NAGS deficiency can be rescued by gene therapy, and suggest that L-arginine binding to the NAGS enzyme is essential for normal ureagenesis.

## Introduction

The urea cycle functions in the liver to protect the central nervous system from the toxic effects of ammonia, a nitrogenous waste product of the catabolism of dietary and cellular proteins. Six urea cycle enzymes and two mitochondrial transporters collectively convert ammonia into urea, which is excreted through the kidneys^1^. Defects in any of the urea cycle enzymes or transporters lead to hyperammonemia, which can damage the brain and could be lethal if left untreated^2^. N-acetylglutamate synthase (NAGS; EC2.3.1.1) is a mitochondrial enzyme that catalyzes formation of N-acetylglutamate (NAG) from glutamate and acetyl coenzyme A^3–5^. NAG is an essential allosteric activator of carbamylphosphate synthetase 1^6–9^ (CPS1; EC 6.3.4.16), the rate-limiting enzyme of the urea cycle^10^. Genetic defects in the *NAGS* gene cause NAGS deficiency (MIM# 237310), which is the only urea cycle disorder that can be easily treated with a drug, N-carbamylglutamate, that binds to and activates CPS1 to fully restore ureagenesis^2^. The NAGS knockout (*Nags*^−/−^) mouse is an animal model of NAGS deficiency that can survive into adulthood and reproduce when treated with N-carbamylglutamate and L-citrulline (NCG + Cit) from birth and develops acute hyperammonemia when this treatment is withdrawn^11^. Because *Nags*^*−/−*^ mice survive with NCG + Cit supplementation, they are ideally suited for studying and optimizing different methods of gene therapy, including adeno associated virus (AAV) based gene transfer. The *Nags*^*−/−*^ mice are also uniquely suitable for studying the function of NAGS in vivo*.*

Human and mouse NAGS proteins appear to be tetramers^12,13^. Each NAGS monomer comprises two structural domains, the C-termina acetyltransferase domain, which binds substrates and catalyzes formation of NAG, and N-terminal amino acid kinase domain, which harbors an L-arginine binding site^13,14^. L-arginine is an allosteric activator of mammalian NAGS^3–5,15,16^. The allosteric effect of L-arginine on NAGS activity changed during evolution; NAGS from fish is partially inhibited by L-arginine^17,18^, while in microorganisms and plants NAGS, the first enzyme of arginine biosynthesis^19,20^, is inhibited by L-arginine^18,19^. Despite this, the L-arginine binding site is conserved in NAGS proteins across phyla. Mutagenesis of amino acids that form L-arginine binding site in bacterial NAGS^18,21^ and corresponding residues in mammalian NAGS^18^ abolished inhibition of mutated bacterial NAGS and activation of mutated mammalian NAGS. Because of the inversion of the allosteric effect of L-arginine on bacterial and mammalian NAGS, amino acids outside of the L-arginine binding site must be responsible for the difference. Random mutagenesis followed by selection for L-arginine insensitive NAGS in *E. coli* as well as comparisons of *Pseudomonas aeruginosa* NAGS and N-acetylglutamyl kinase protein sequences and mutagenesis studies identified several amino acids in both domains that mediate inhibition of bacterial NAGS by L-arginine^21,22^. Additionally, the length and sequence of the linker connecting amino acid kinase and acetyltransferase domains regulate the allosteric effect of L-arginine on NAGS^23,24^.

Allosteric activation of mammalian NAGS by L-arginine suggests that it could regulate the rate of urea production through modulation of NAGS activity to supply variable amounts of NAG for controlled activation of CPS1. Correlations between dietary protein intake, L-arginine concentration in portal blood, hepatic NAG concentration, and excretion of urea implicate L-arginine in regulation of ureagenesis^25–31^. Moreover, the magnitude of murine NAGS activation by L-arginine in the liver mitochondria depends on the dietary protein intake and animal’s satiety^4,5,30^. The first step towards resolving the role of L-arginine in the production of NAG and regulation of ureagenesis is to determine whether it becomes impaired when NAGS cannot bind L-arginine.

In this study, we used AAV-based gene transfer to correct NAGS deficiency in Nags^−/−^ mice; we determined the dose of AAV vectors needed to prevent hyperammonemia after withdrawal of NCG + Cit supplementation from the treated *Nags*^*−/−*^ mice, and measured expression of NAGS mRNA and protein in their livers. To analyze the behavior of Nags^−/−^ mice, we employed either a Home Cage Behavioral system or voluntary wheel to quantify their activity during a healthy, treated state, and as they developed hyperammonemia following the withdrawal of NCG + Cit. We then used this approach to determine whether E354A mutant mNAGS, that is not activated by L-arginine^18^, can complement NAGS deficiency in the *Nags*^*−/−*^ mice, and evaluated their plasma ammonia and activity on the voluntary wheel after withdrawal of NCG + Cit supplementation. Finally, we characterized human NAGS protein that cannot bind L-arginine that was identified in a patient with NAGS deficiency.

## Results

### Non-invasive tracking of hyperammonemia

We used either the Home Cage Scan behavioral monitoring system or voluntary wheel to chronologically assess behavioral and activity changes that occured during the development of acute hyperammonemia in the *Nags*^−/−^ mice. Ammonia concentration in the blood of *Nags*^*−/−*^ mice started to rise after withdrawal of the NCG + Cit supplementation and continued during 24 h of behavior monitoring. Six behaviors were monitored with the Home Cage Scan system: eating, drinking, hanging, walking, grooming, and rearing up; we also determined whether time to cessation of each activity differed depending on the withdrawal of the NCG + Cit treatment at either 4 PM or 10 AM. Mouse activity was recorded for 48 h.

**Figure 1:**
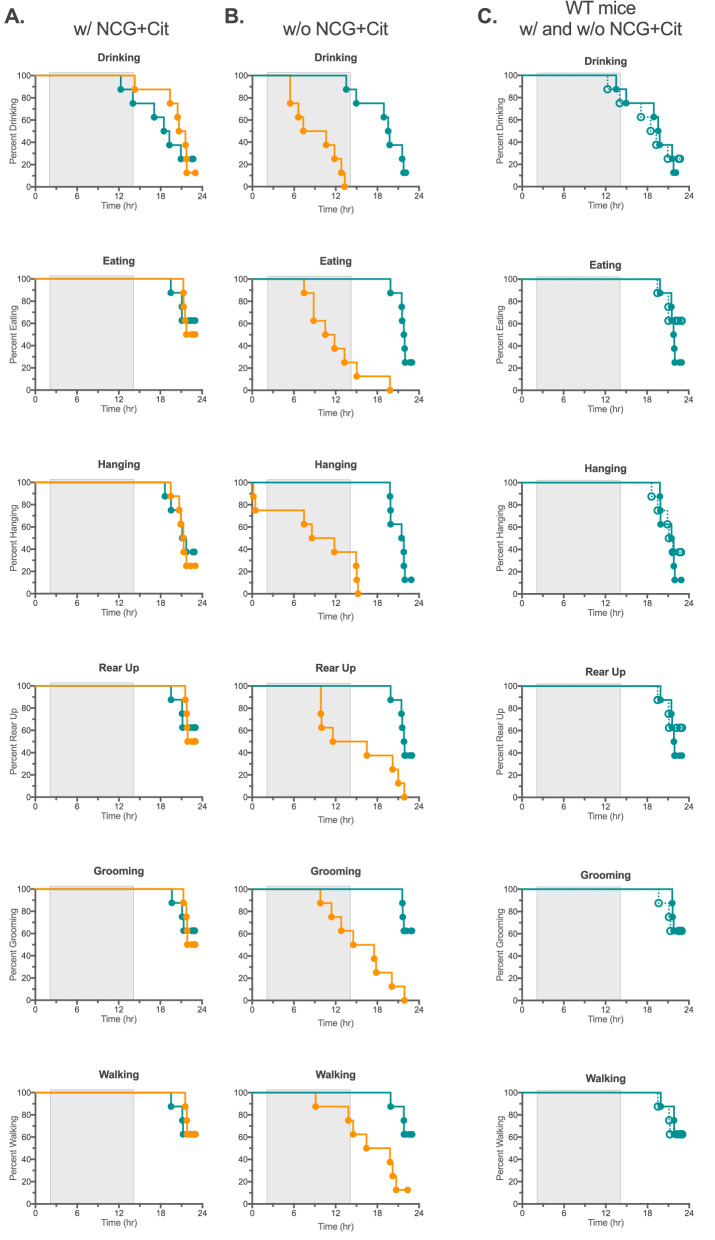
Differing behaviors of *Nags*^*−/−*^ (orange) and *Nags*^+*/*+^ (teal) mice with respect to cessation of drinking, eating, hanging, rearing up, grooming, and walking (**A**) during the 24 h before withdrawal of NCG + Cit supplementation and (**B**) in the 24 h period after withdrawal of NCG + Cit supplementation at 4 PM. The same results are shown in (**C**) for wild-type mice before (solid) and after (hatched) withdrawal of NCG + Cit supplementation. Shaded rectangles indicate the period of darkness between 6 PM and 6 AM.

During the first 24 h period mice were treated with NCG + Cit. There was no significant difference between *Nags*^−/−^ and wild-type mice in any of the measured behaviors (Fig. 1A). The *Nags*^−/−^ mice started to cease drinking, eating, and hanging behaviors between 10 min. and 5 h after the 4 PM withdrawal of NCG + Cit treatment (Fig. 1B). The cessation of rearing, walking, and grooming occurred between 9 and 22 h after withdrawal of the NCG + Cit treatment at 4PM (Fig. 1B). The behavior of the wild-type mice was similar before and after the 4 PM withdrawal of the NCG + Cit treatment (Fig. 1C).

**Figure 2:**
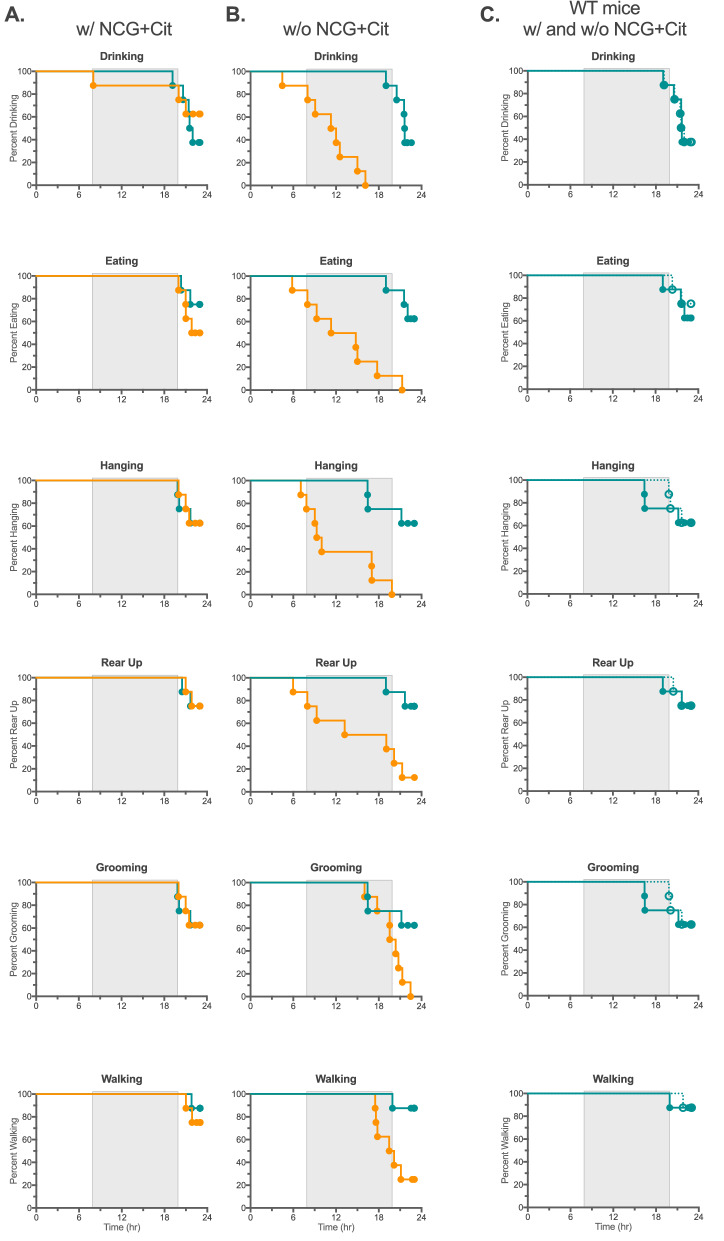
Different behaviors of the *Nags*^*−/−*^ (orange) and *Nags*^+*/*+^ (teal) mice with respect to cessation of drinking, eating, hanging, rearing up, grooming, and walking (**A**) during 24 h before withdrawal of NCG + Cit supplementation and (**B**) in the 24 h period after withdrawal of NCG + Cit supplementation at 10 AM. The same results are shown in (**C**) for wild-type mice before (solid) and after (hatched) withdrawal of NCG + Cit supplementation. Shaded rectangles indicate the period of darkness between 6 PM and 6 AM.

We thought that the timing of NCG + Cit withdrawal might affect the onset of symptoms related to acute hyperammonemia because mice are not active during the daytime and may not drink a significant amount of water during the light part compared to dark part of the cycle. To account for circadian rhythm effects, the experiment was repeated with the NCG + Cit withdrawal at 10 AM. As in the previous experiment, the behaviors of the *Nags*^−/−^ and wild-type mice were similar before withdrawal of the NCG + Cit treatment (Fig. 2A). Between 4.5 and 17.5 h after NCG + Cit withdrawal at 10 AM, the *Nags*^−/−^ mice started to cease drinking, hanging, eating, rearing up, grooming, and walking (Fig. 2B). Drinking and hanging were the first two activities to cease, followed by eating and rearing up; grooming and walking continued until 17–24 h after withdrawal of NCG + Cit (Fig. 2B). The wild type mice exhibited similar activity levels before and after withdrawal of NCG + Cit, indicating that NCG and L-citrulline have no effect on the activity of healthy mice (Fig. 1C). Although the Home Cage Scan system provides detailed information about multiple mouse behaviors, it has low throughput and we sought to develop a higher throughput method for evaluation of the *NAGS*^*−/−*^ behavior after withdrawal of the rescue chemicals.

### Rescue of Nags^−/−^ phenotype with AAV gene therapy

NCG is an FDA approved drug for *NAGS* deficient patients; however, the life-long therapy is expensive. A more efficient treatment would be transfer of the NAGS gene into hepatocytes. Pre-clinical studies have demonstrated the success of correcting ornithine transcarbamylase deficiency in mice using AAV gene therapy^32^. Therefore, we evaluated whether AAV-based gene transfer of the mouse NAGS (mNAGS) can rescue *Nags*^−/−^ mice from hyperammonemia after withdrawal of the NCG treatment, and whether the *Nags* gene promoter can be used to control expression of the delivered *Nags* gene.

**Figure 3:**
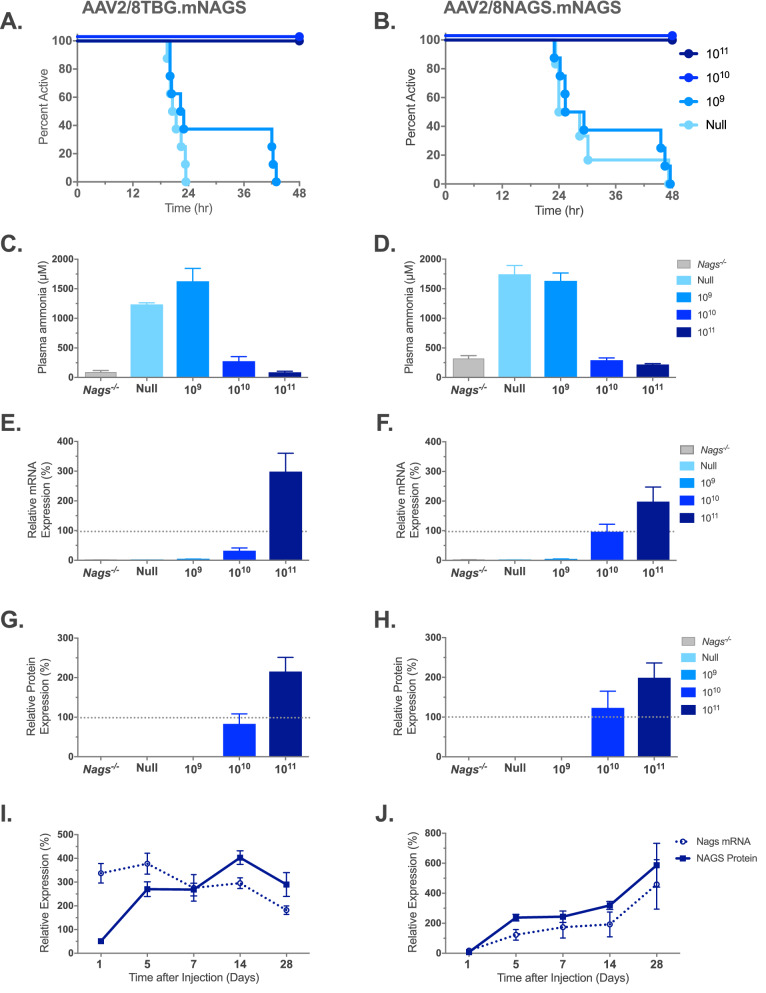
Phenotype of the *Nags*^*−/−*^ mice after expression of the NAGS gene from the AAV2/8TBG.mNAGS vector (left panels), or the AAV2/8NAGS.mNAGS vector (right panels). (**A**,**B**) Duration of activity on the voluntary wheel. (**C**,**D**) Plasma ammonia concentrations. (**E–H**) Abundance of the *Nags* mRNA (**E**,**F**) and NAGS protein (**G**,**H**) in the liver. (**I**,**J**) Changes in the abundance of *Nags* mRNA and NAGS protein in the liver after a single injection of 10^11^ viral particles. Each column in panels E–H represents a mean and its associated SEM in n = 8 mice; each data point in panels I and J represents a mean and its associated SEM in n = 6 mice where 100% is set as the mean value for four *Nags*^+*/*+^ mice in panels E–H. Abundance of the *Nags* mRNA and NAGS protein in injected and *Nags*^+*/*+^ mice were normalized to the abundance of 18S rRNA and vinculin, respectively.

The number of AAV viral particles needed for the rescue of *Nags*^−/−^ mice and the corresponding expression levels of mNAGS mRNA and protein were determined first. Expression of mNAGS was controlled either by the human thyroxine-binding-globulin (*TBG*) or natural mouse *Nags* promoter. To determine the dose of vector needed for the rescue of *Nags*^−/−^ mice, 6–8 weeks old animals were injected with 10^9^, 10^10^, or 10^11^ viral particles of either AAV2/8.TBG.mNAGS or AAV2/8.NAGS.mNAGS vector. Control mice were injected with 10^11^ particles of the AAV2/8TBG vector without the mNAGS coding sequence insert (null vector); these mice were expected to develop acute hyperammonemia and stop running on the wheel. Five days after injection, mice were transferred into cages with voluntary wheels and, after an acclimation period, their activity on the wheel was monitored for 48 h before and after withdrawal of NCG from the drinking water. L-citrulline supplementation continued throughout the experiment to ensure endogenous biosynthesis of arginine^33^. All *Nags*^−/−^ mice injected with 10^10^ and 10^11^ particles of either vector appeared healthy and were active for 48 h after withdrawal of NCG from the drinking water, indicating complete rescue by these two doses of viral particles (Fig. 3A,B). The lowest dose of 10^9^ viral particles of either vector did not rescue *Nags*^−/−^ mice, although some did cease activity on the wheel close to 48 h after withdrawal of NCG (Fig. 3A,B).

Plasma ammonia and expression of *Nags* mRNA and mNAGS protein were measured after control mice injected with the null vector, and mice injected with 10^9^ virus particles stopped running on the wheel. The same measurements were done at the end of the 48 h activity monitoring period in the mice that received either 10^10^ or 10^11^ viral particles, and were rescued by gene transfer. Plasma ammonia in *Nags*^−/−^ mice supplemented with NCG + Cit was used as control. Mice injected with the null vector and mice injected with 10^9^ particles of either AAV2/8TBG.mNAGS or AAV2/8NAGS.mNAGS developed severe hyperammonemia after they stopped running on the wheel, while plasma ammonia was similar to the baseline in mice injected with 10^10^ and 10^11^ doses of either vector (Fig. 3C,D).

**Figure 4:**
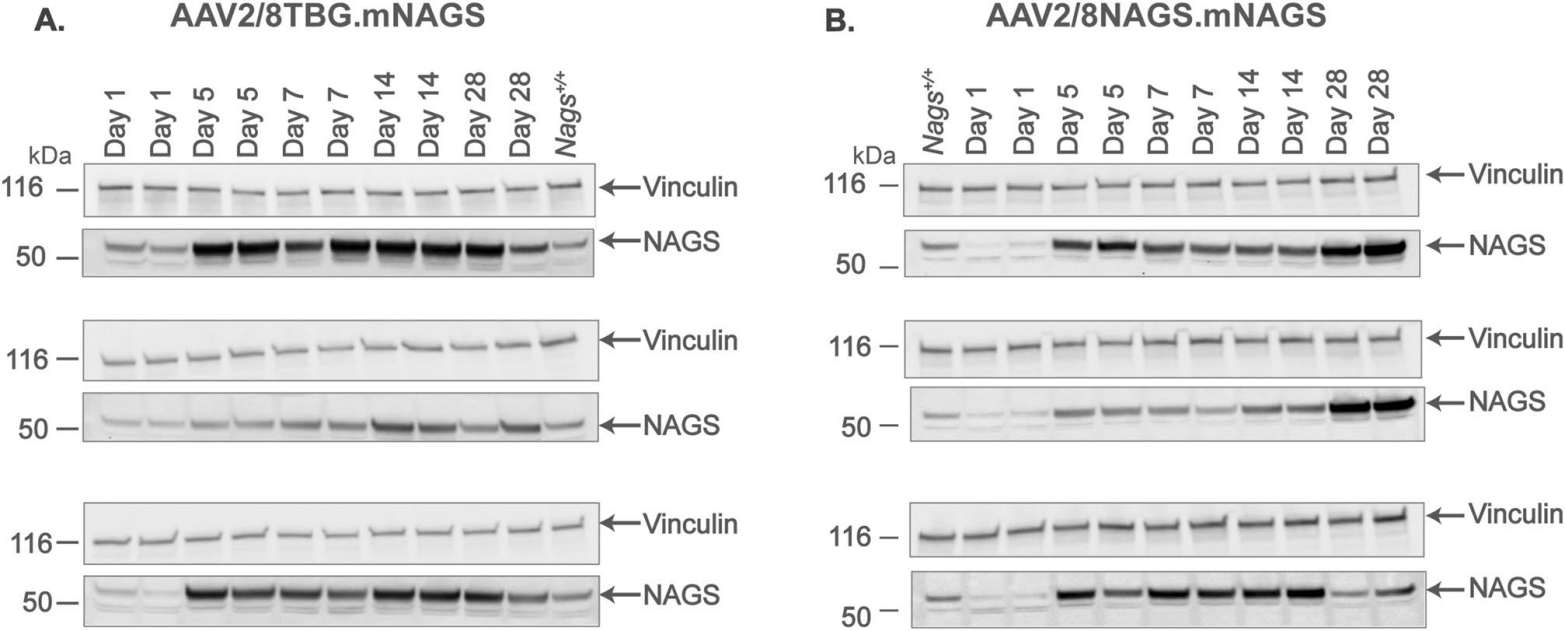
NAGS protein in the livers of *Nags*^*−/−*^ mice injected with increasing doses of either AAV2/8TBG.mNAGS (**A**) or AAV2/8NAGS.mNAGS (**B**) vector. AAV2/8TBGnull (Null) vector was injected as a control. Vinculin was used as a loading control. Full-length immunoblots are presented in Supplementary Fig. S1.

Real-time PCR was used to determine the abundance of *Nags* mRNA and immunoblotting was used to determine the abundance of NAGS protein. The *Nags*^−/−^ mice supplemented with NCG + Cit and wild-type mice were used as negative and positive controls for mRNA and protein measurements. The *Nags* mRNA and mNAGS protein were undetectable in the livers of *Nags*^−/−^ mice injected with the null vector (Fig. 3E–H). The abundance of NAGS mRNA was below 5% of the wild-type levels in mice injected with 10^9^ particles of either AAV2/8TBG.mNAGS or AAV2/8NAGS.mNAGS vector (Fig. 3E,F; Table 1); NAGS protein was undetectable in these animals (Figs. 3G,H, 4; Table 1). The average abundance of *Nags* mRNA in mice injected with 10^10^ AAV2/8TBG.mNAGS particles was below wild-type levels (Fig. 3E; Table 1), but the abundance of NAGS protein in these mice was similar to wild-type levels (Figs. 3G, 4A; Table 1). Relative abundance of *Nags* mRNA and mNAGS protein in the *Nags*^*−/−*^ mice injected with 10^11^ particles of the AAV2/8TBG.mNAGS vector were about twice as high as *Nags* mRNA and mNAGS protein levels in wild-type mice (Figs. 3E,G, 4A; Table 1). Abundance of *Nags* mRNA and mNAGS protein were similar in wild-type mice and *Nags*^-/-^ mice injected with 10^10^ viral particles of AAB2/8NAGS.mNAGS, while NAGS^−/−^ mice injected with the highest dose of the AAV2/8NAGS.mNAGS vector had about two-fold higher levels of *Nags* mRNA and mNAGS protein than wild-type mice (Figs. 3F,H, 4B; Table 1).

**Table 1:** Relative expression of *Nags* mRNA and NAGS protein in *Nags*^*−/−*^ mice injected with increasing doses of AAV2/8TBG.mNAGS and AAV2/8NAGS.mNAGS viral vectors.

	AAV2/8TBG.mNAGS			AAB2/8NAGS.mNAGS		
	10^9^	10^10^	10^11^	10^9^	10^10^	10^11^
Relative mRNA expression (%)^a^	3.0 ± 0.9^b^	32.2 ± 9.2	298.4 ± 62.2	3.7 ± 0.9	96.4 ± 25.5	198.3 ± 49.3
Relative protein expression (%)^a^	nd^c^	83.1 ± 25.2	215.3 ± 36.1	nd	123.0 ± 42.2	198.9 ± 37.2

^a^Abundance of *Nags* mRNA and protein in wild type mice was set as 100%.

^b^Values represent mean ± SEM, n = 8.

^c^Not detectable.

**Figure 5:**
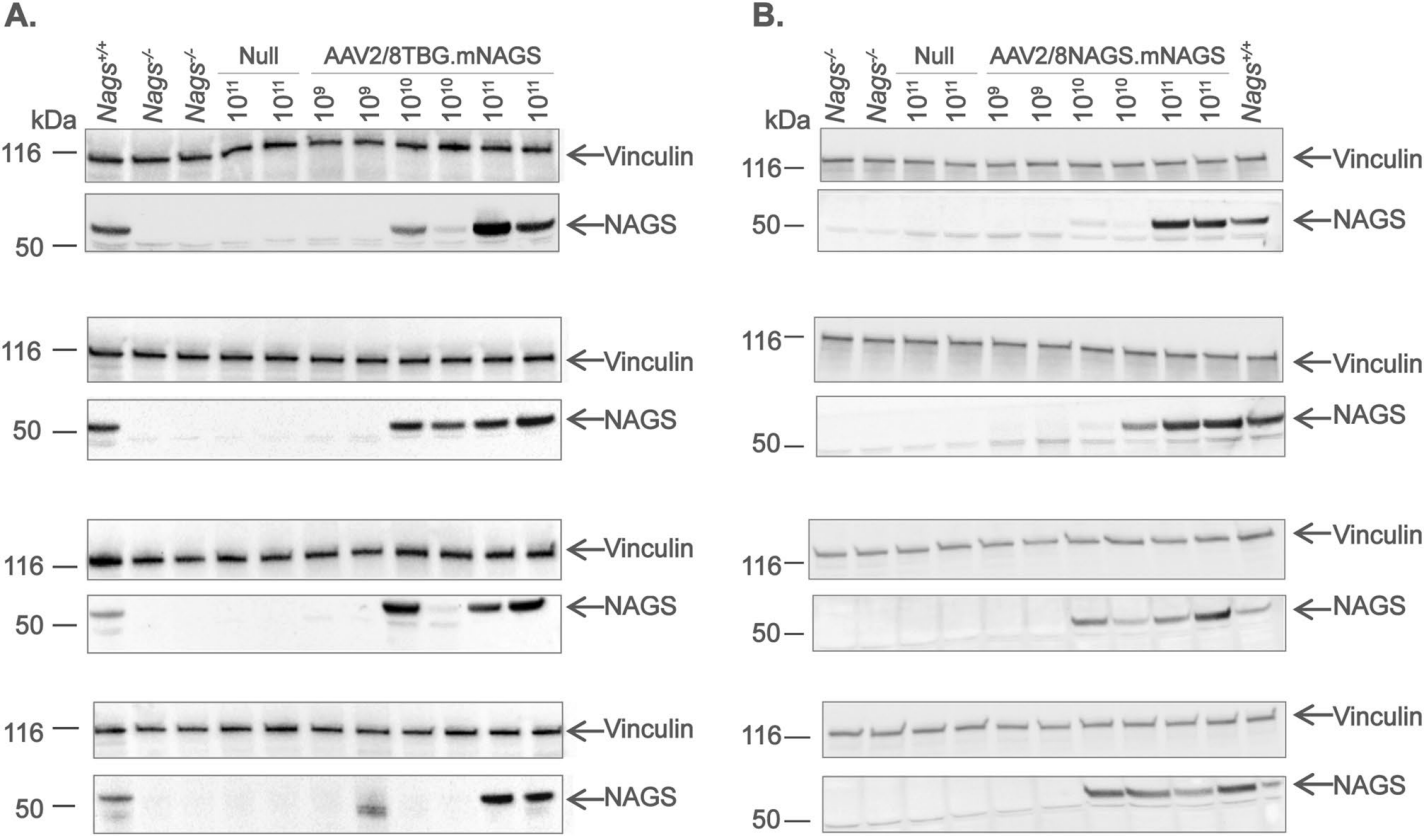
NAGS protein in the livers of *Nags*^*−/−*^ mice after single injection with 10^11^ particles of either AAV2/8TBG.mNAGS (**A**) or AAV2/8NAGS.mNAGS (**B**) vector. Vinculin was used as a loading control. Full-length immunoblots are presented in Supplementary Fig. S2.

We also determined the abundance of *Nags* mRNA and mNAGS protein 1, 5, 7, 14 and 28 days after a single injection of 10^11^ particles of either AAV2/8TBG.mNAGS or AAV2/8NAGS.mNAGS vector. In mice injected with AAV2/8TBG.mNAGS vector *Nags* mRNA and mNAGS protein were expressed one day after injection (Figs. 3I, 5A and S2A). The abundance of *Nags* mRNA peaked on the fifth day after injection and decreased thereafter, while mNAGS protein abundance peaked on day 14 and was lower 28 days after injection (Figs. 3I, 5A). Expression patterns of *Nags* mRNA and mNAGS protein were different in mice injected with the AAV2/8NAGS.mNAGS vector. In these mice average abundance of *Nags* mRNA and mNAGS protein was less than 20% of the *Nags* mRNA and mNAGS abundance in wild-type mice one day after injection of AAV2/8-NAGS.mNAGS and increased thereafter (Figs. 3J, 5B).

### *Functional testing of arginine-insensitive NAGS *in vivo

**Figure 6:**
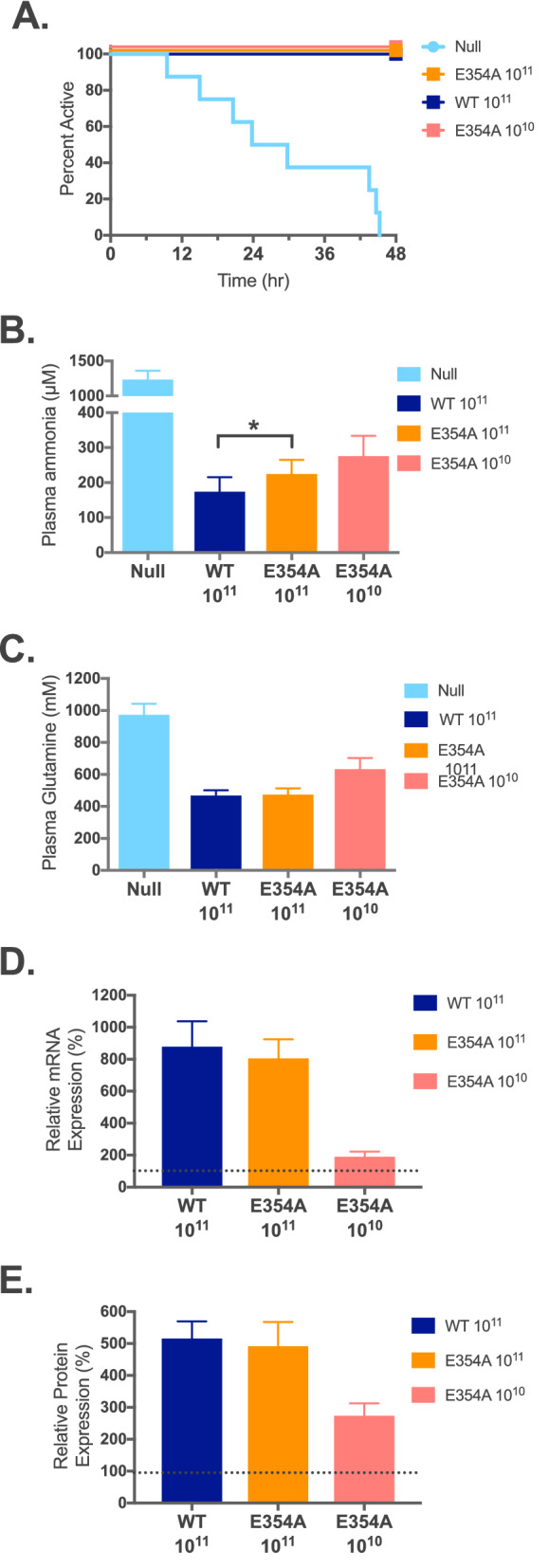
Phenotype of the *Nags*^*−/−*^ mice that received AAV2/8NAGS.mNAGS-E354A gene therapy. Duration of activity on the voluntary wheel (**A**), plasma ammonia (**B**) and glutamine (**C**) concentrations, abundance of the liver NAGS mRNA (**D**) and protein (**E**) after withdrawal of NCG. Each column represents a mean and its associated SEM of measurements in n = 8 animals; 100% is the mean value for four *Nags*^+*/*+^ mice.

**Figure 7:**
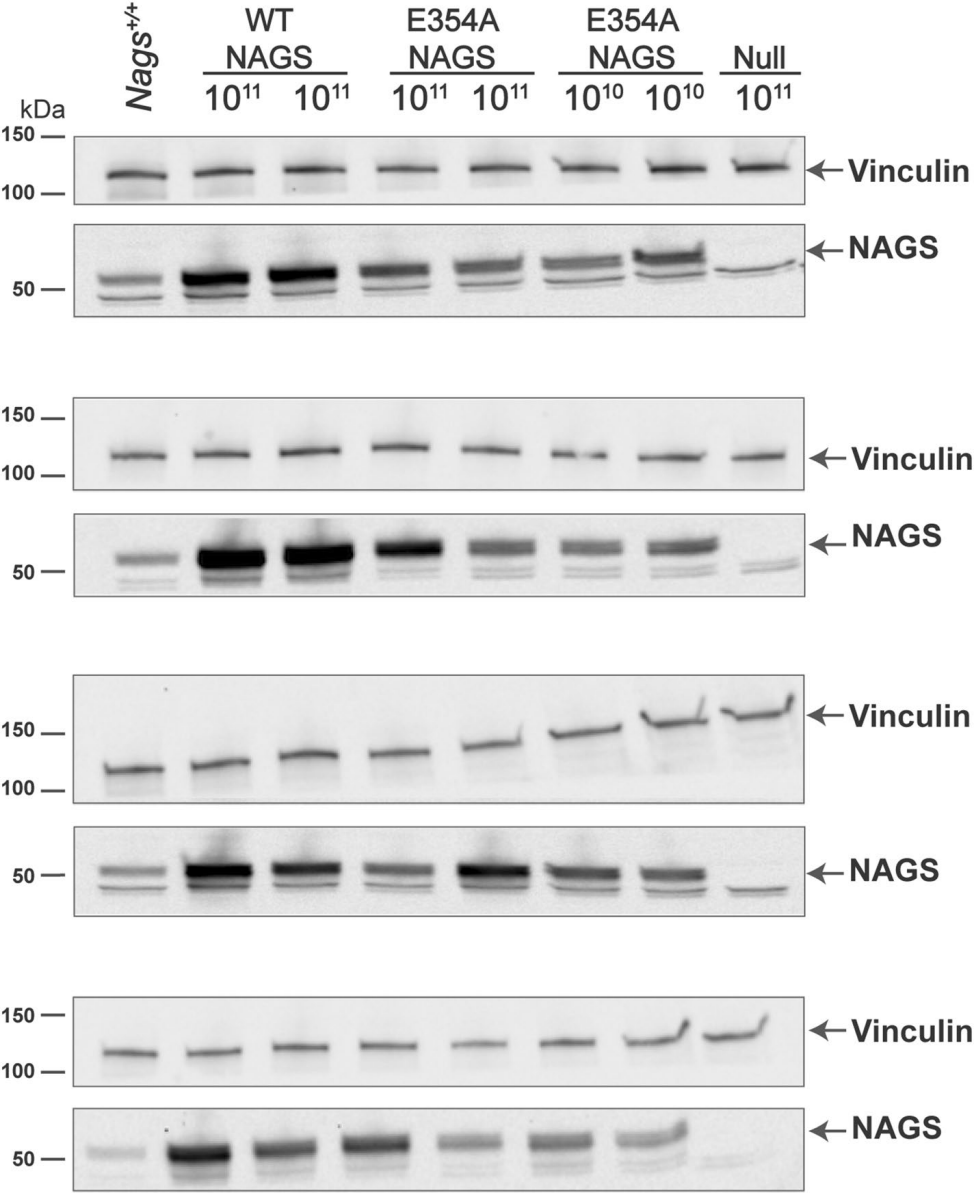
NAGS protein in the livers of *Nags*^*−/−*^ mice injected with 10^11^ viral particles of the AAV2/8NAGS.mNAGS vector (WT NAGS lanes), with either 10^11^ or 10^10^ viral particles of the AAV2/8NAGS.E354A-mNAGS vector (E354A NAGS lanes), or with 10^11^ viral particles of the AAV2/8TBGnull vector (Null). Vinculin was used as a loading control. Full-length immunoblots are presented in Supplementary Fig. S3.

To determine the contribution of L-arginine to NAGS function, we used the above AAV-based gene transfer and activity measurements on the voluntary wheel to determine whether the E354A arginine insensitive mutant mNAGS^18^ can rescue *Nags*^−/−^ mice from hyperammonemia. The *Nags*^−/−^ mice (6–8 weeks old) were injected with either 10^10^ or 10^11^ viral particles of the AAV2/8NAGS.mNAGS-E354A vector. Five days after injection the mice were placed in cages with a voluntary wheel, allowed two days to acclimate to the new environment, and their activity was recorded for 48 h before and after withdrawal of NCG from their drinking water. Mice injected with 10^11^ viral particles of either AAV2/8NAGS.mNAGS (wild type) or AAV2/8TBGnull were a positive and negative control, respectively. The mice that received either AAV2/8NAGS.mNAGS-E354A or AAV2/8NAGS.mNAGS remained active for 48 h after withdrawal of NCG, while the activity of mice injected with the null vector ceased between 10 and 45 h post NCG withdrawal (Fig. 6A). Although mice that received 10^11^ vector particles encoding E354A mutant NAGS appeared healthy and remained active for 48 h after withdrawal of NCG, their plasma ammonia was elevated (Fig. 6B) and plasma ammonia was even higher in mice that received 10^10^ particles of the AAV2/8NAGS.mNAGS-E354A vector. There was a trend toward higher plasma glutamine levels in mice that received 10^10^ viral particles of the AAV2/8NAGS.mNAGS-E354A vector compared to the 10^11^ dose (Fig. 6C). Measurements of the mRNA and protein abundance revealed no difference in expression levels between the wild-type and E354A mutant mNAGS in the livers of *Nags*^−/−^ mice that were injected with the same dose of either AAV2/8NAGS.mNAGS or AAV2/8NAGS.mNAGS-E354A (Figs. 6D,E, 7 and S3). This suggests that E354A mutant NAGS cannot produce sufficient amount of NAG needed for conversion of all nitrogenous waste into urea.

**Figure 8:**
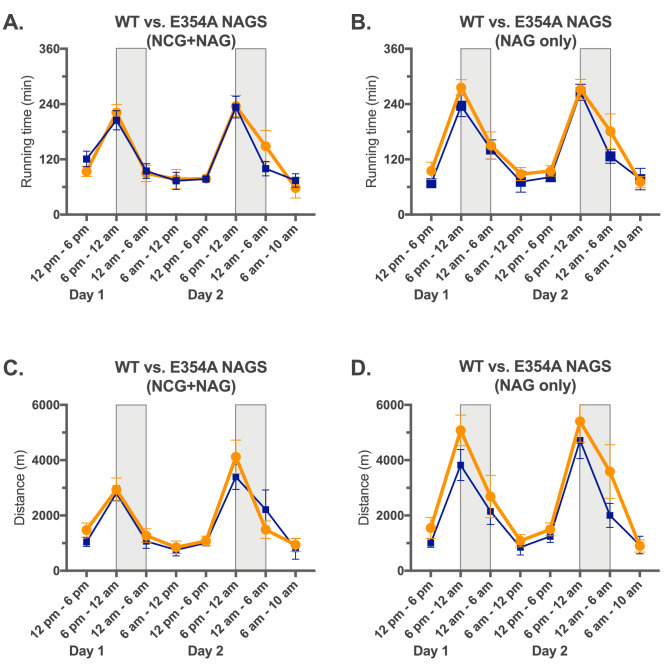
Behavior of *Nags*^−/−^ mice that received the same dose of either AAV2/8NAGS.mNAGS-E354A or AAV2/8NAGS.mNAGS gene therapy. (**A**) Average running time during 6 h epochs before withdrawal of NCG supplementation. (**B**) Average running time during 6 h epochs after withdrawal of NCG supplementation. (**C**) Average running distance during 6 h epochs before withdrawal of NCG supplementation. (**D**) Average running distance during 6 h epochs after withdrawal of NCG supplementation. Orange—AAV2/8NAGS.mNAGS-E354A. Blue—AAV2/8NAGS.mNAGS. Shaded rectangles indicate nighttime when mice are more active. (NCG + NAG) indicates that both NCG and NAGS are present in the livers of experimental animals. (NAG only) indicates that NCG supplementation has been withdrawn and NAG is produced by the mNAGS expressed from the AAV-vector while NCG is absent from the livers of experimental animals. Shaded rectangles indicate periods of darkness between 6 PM and 6 AM.

We analyzed activity of the *Nags*^−/−^ mice that were injected with 10^11^ viral particles of either AAV2/8NAGS.mNAGS or AAV2/8NAGS.mNAGS-E354A to determine whether hyperammonemia affected their behavior on the voluntary wheel. The 48 h before and after withdrawal of NCG supplementation were divided into 6 h epochs followed by calculation of the running time and distance for each mouse during each epoch. Before withdrawal of NCG supplementation, both NCG and NAG should be present in the livers to activate CPS1, while after withdrawal of NCG, CPS1 should be activated by the NAG produced by either E354A or wild-type mNAGS, which were introduced by gene transfer. Running time of the *Nags*^−/−^ mice that received either wild-type or E354A mNAGS did not differ before withdrawal of the NCG supplementation (Fig. 8A); after withdrawal of NCG, there was a trend (*p* = 0.06) towards longer running time for mice that received E354A mNAGS (Fig. 8B). Running distance was similar for mice that received either wild-type or E354A mNAGS before withdrawal of NCG (Fig. 8C), while after NCG withdrawal the distance run by the mice that received the E354A mNAGS was longer than the distance run by the mice that received wild-type mNAGS (Fig. 8D). During nighttime, when mice are most active, the average distances run by animals expressing E354A mutant mNAGS were between 14 and 79% longer than distances run by mice expressing wild-type mNAGS (Fig. 8D). This suggests that mice may run faster when they are hyperammonemic. To confirm this, we determined the length of each running session on the wheel, the distance a mouse run during that session, and its velocity for the 6 h epochs after withdrawal of NCG supplementation. At nighttime, *Nags*^−/−^ mice that received E354A mutant mNAGS ran longer distance because they ran faster and longer than mice that received wild-type mNAGS (Fig. S4).

### NAGS deficiency due to arginine-insensitive NAGS

**Figure 9:**
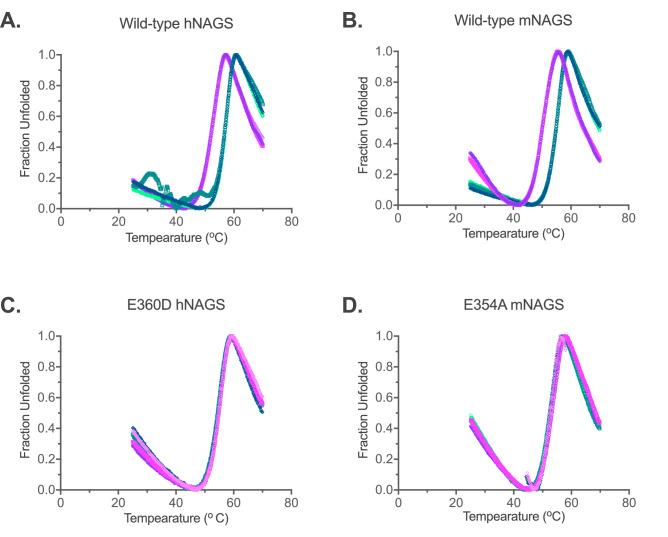
Thermal stability of wild-type human (**A**) and mouse (**B**) NAGS, E360D human NAGS (**C**), and E354D mouse NAGS (**D**) in the presence of either L-arginine (purple) or D-arginine (teal). Different shades of purple and teal represent technical replicates of thermal unfolding.

The clinical relevance of arginine-insensitive NAGS was confirmed when a patient with adult-onset partial NAGS deficiency was found to be a compound heterozygous for two sequence variants, NM_153006.2:c.1080G > T (NP_694551.1:p.E360D) and NM_153006.2:c.426 + 3G > C, while we were testing the function of the arginine insensitive E354A mutant mNAGS in mice. Functional testing of the c.426 + 3G > C sequence variant indicated that it could diminish NAGS expression, presumably due to impaired mRNA splicing (Fig. S5). The p.E360D amino acid substitution affects a glutamate residue in human NAGS (hNAGS) that corresponds to the E354 in mNAGS^18,34^. To determine the functional consequences of this substitution, the E360D and E354D mutations were engineered into hNAGS and mNAGS, respectively, followed by overexpression in *E. coli* and purification of mutant and wild-type recombinant hNAGS and mNAGS (Fig. S6). We tested whether the enzymatic activity of the four recombinant proteins increases in the presence of L-arginine and whether they can bind L-arginine. The results revealed that the enzymatic activities of the wild-type hNAGS and mNAGS doubled in the presence of 1 mM L-arginine, while the activities of the E360D hNAGS and E354D mNAGS did not change in the presence of 1 mM L-arginine (Table 2). A Thermofluor^®^ assay was used to determine the effect of L-arginine on thermal unfolding of wild-type and mutant recombinant hNAGS and mNAGS; D-arginine, which does not bind to mNAGS^12^, was used as a control. The Tm (temperature at which half of the protein molecules are unfolded), of both hNAGS and mNAGS increased by 4.60 ± 0.05 °C and 4.70 ± 0.12 °C (mean ± SD, n = 3), respectively, in the presence of L-arginine (Fig. 9A,B), while the Tm of the E360D hNAGS and E354D mNAGS remained the same in the presence of L- and D-arginine (Fig. 9C,D). This suggests that these mutant hNAGS and mNAGS proteins cannot bind L-arginine, explaining the absence of its effect on NAGS enzymatic activity.

**Table 2:** Effect of L-arginine on enzymatic activities of either wild-type or mutant human and mouse NAGS.

Protein	NAGS specific activity (µmoles min^−1^ mg^–1^)	
		+ 1 mM L-Arg
Wild-type human NAGS	7.17 ± 1.02^a^	13.98 ± 1.95
E360D human NAGS	10.15 ± 0.10	10.05 ± 0.20
Wild-type mouse NAGS	13.29 ± 0.08	23.94 ± 0.37
E354D mouse NAGS	8.28 ± 0.13	8.24 ± 0.06

^a^Enzymatic activities are averages of three measurements and the associated standard errors.

## Discussion

In this study we have shown that behavior of hyperammonemic mice mimics symptoms of hyperammonemic patients^2^ and that AAV-based gene therapy can be used to correct NAGS deficiency in the *Nags*^*−/−*^ mice. We have shown that both vectors with strong TBG promoter and endogenous *Nags* promoter enable expression of similar amounts of NAGS protein in the livers of treated mice. Although we have shown either similar or about two-fold higher expression of NAGS protein in the *Nags*^*−/−*^ mice that received two different doses of AAV vectors, our measurements represent average NAGS abundance in all hepatocytes, and may not reflect abundance of NAGS in periportal hepatocytes that express other urea cycle genes.

The results presented here provide direct evidence that binding of L-arginine to mammalian NAGS is required for its normal function. The *Nags*^−/−^ mice that expressed the E354A mutant mNAGS, which is not activated by L-arginine^18^, were hyperammonemic despite similar expression levels of the mutant and wild-type *Nags* mRNA and mNAGS protein (Fig. 6). Specific activities of recombinant E354A and wild-type mNAGS were similar in the absence of L-arginine^18^. Assuming that the specific activities of both E354A and wild-type mNAGS are similar in vitro and in vivo, E354A mNAGS should be able to produce enough NAG for normal ureagenesis. Hyperammonemia in *Nags*^*−/−*^ mice expressing E354A mNAGS suggests that production of NAG in these mice is not sufficient for activation of CPS1 and normal urea production. Although it is possible that intramitochondrial arginine concentration was higher in our experimental animals due to continuous citrulline supplementation, it was insufficient to overcome the inability of E354A mutant NAGS to bind arginine because our mice expressing E354A mNAGS had higher plasma ammonia than mice expressing wild type mNAGS. The two groups of *Nags*^*−/−*^ mice expressing E354A mutant mNAGS had 1.3 and 1.6-fold higher concentration of plasma ammonia than the *Nags*^*−/−*^ mice expressing wild type mNAGS. Although these increases in the concentration of plasma ammonia may seem small they are comparable increases in plasma ammonia concentrations that trigger symptoms of hyperammonemia in patients with *NAGS* deficiency^35,36^.

Although doubling of human and mouse NAGS enzymatic activities in the presence of L-arginine^37^ may appear insufficient to accommodate the need for increased ureagenesis in situations of increased ammonia load, increased production of NAG is amplified through continuous activation of additional CPS1 molecules. Earlier studies of activation of murine NAGS by L-arginine in the liver mitochondria have shown different magnitudes of NAGS activation depending on the dietary protein intake and time after a meal^4,5,30^, but did not provide a mechanism for this effect of L-arginine on NAGS activity. One explanation could be that NAGS is activated by L-arginine resulting from the breakdown of dietary protein, but other factors such as post-translational modifications could be contributing to this phenomenon. Acetylation, succinylation, malonylation or glutarylation of the NAGS lysine residues could affect NAGS activation by L-arginine; removal of these post-translational modification by mitochondrial sirtuins SIRT 3 and SIRT5^38–40^ could provide an explanation for variable activation of NAGS by L-arginine.

The importance of L-arginine binding to NAGS for normal ureagenesis was confirmed by the case of a hyperammonemic patient found to have a E360D substitution in NAGS; this mutated protein neither binds nor is activated by L-arginine, suggesting that inability to bind L-arginine impairs the function of human NAGS, leading to decreased ureagenesis and hyperammonemia. Another possibility is that the inability to bind L-arginine has a dominant negative effect on the function of NAGS heterotetramers^13^ that consist of wild-type and mutant subunits in the patient with NAGS deficiency.

The voluntary wheel was used to elucidate the biological consequences of arginine-insensitive NAGS on urea cycle function and hyperammonemia development. The hyperammonemic *Nags*^−/−^ mice that expressed E354A mutant mNAGS and the *Nags*^−/−^ mice that expressed wild-type NAGS had different patterns of activity on the voluntary wheel. Hyperammonemic mice that received E354A mutant NAGS ran longer distances on the voluntary wheel during nighttime than the mice that received the same dose of wild type mNAGS. This was because hyperammonemic mice ran faster than the mice that received wild-type NAGS and both groups of mice spent similar amount of time running on the voluntary wheel. This suggests that hyperammonemic mice may be hyperactive, similar to patients with urea cycle disorders who can have symptoms of attention deficit hyperactivity disorder^41,42^.

The phenotype of *Nags*^*−/−*^ mice experiencing chronic hyperammonemia was different from the phenotype of *Nags*^*−/−*^ mice with acute hyperammonemia. The *Nags*^*−/−*^ mice that received gene therapy with E354A mutant mNAGS had chronically elevated blood ammonia concentration, remained active for the duration of the experiment (Fig. 6A), and their pattern and level of activity was different from that of mice with normal blood ammonia (Figs. 8 and S4). *Nags*^*−/−*^ mice experiencing acute hyperammonemia had a phenotype similar to some symptoms in patients experiencing hyperammonemic episodes including loss of appetite, decreased activity, and coma^2^.

In mammals and other animals that detoxify ammonia via the urea cycle, the function of NAGS is to provide a cofactor for either CPS1 or CPS3. However, in microbes and plants, NAGS catalyzes the first reaction of L-arginine biosynthesis^19,20,43–45^. The effect of L-arginine on NAGS enzymatic activity changed during evolution; L-arginine acts as a negative feedback signal in organisms with L-arginine biosynthesis, while it partially inhibits NAGS activity in fish and activates NAGS in mammals^18^. Despite these differences, the binding site for L-arginine remains conserved in all NAGS enzymes^18,21^. While feedback inhibition of microbial and plant NAGS proteins provides the obvious benefit of preventing unnecessary consumption of precursors and energy when exogenous L-arginine is abundant, the benefits of binding and activation of mammalian NAGS by L-arginine are less clear. One possibility is that activation of mammalian NAGS by L-arginine together with activation of CPS1 by NAG creates a double positive feedback loop that enables sensitive and robust regulation of ammonia detoxification via ureagenesis and effective protection of the central nervous system from ammonia toxicity^18,46^. Alternatively, L-arginine and its binding to mammalian NAGS could be part of a nitrogen load sensing mechanism that adjusts the abundance of urea cycle enzymes in response to increased protein catabolism due to either high dietary protein intake or degradation of cellular proteins. We will be able to test these hypotheses by combining biochemical testing with monitoring of activity and behavior of *Nags*^−/−^ mice either on the voluntary wheel or in the Home Cage system.

## Materials and methods

### Animal husbandry and genotyping

Animals were housed at Children's National Medical Center and the Veterans Affairs Animal Research Facility and all protocols were approved by The Children’s National Medical Center and V.A. IACUC, Washington, DC. All methods and procedures were performed in accordance with the relevant regulations and ARRIVE guidelines.

Mice were checked daily for signs of distress and were maintained on a 12:12 h light–dark cycle in a low-stress environment (22 °C, 50% humidity and low noise) and given food and water ad libitum. The maintenance of the *Nags*^−/−^ mouse colony has been previously described^11^. For the purposes of these studies, wild type mice were given NCG + Cit supplementation for a 2-week period before the experiments were conducted.

Genomic DNA was isolated either from tail or ear punch biopsy using Gentra Puregene Tissue kit (Qiagen, Valencia, CA) according to manufacturer’s instructions. Mice were genotyped either as described previously^11^ or using multiplex PCR with primers Exon6F: 5′-CAG CTT TAG GAG GAC AGG AGA G-3′, Exon6R: 5′-CTT AGA GAC ACA GAC CAG GAG TTA G-3′, NEO-F: 5′-TGC TCC TGC CGA GAA AGT ATC CAT CAT GGC-3′ and NEO-R: 5′-CGC CAA GCT CTT CAG CAA TAT CAC GGG TAG-3′. The following amplification conditions were used: 2 min. initial denaturation at 95 °C, followed by 25 cycles of 15 s. denaturation at 95 °C, 20 s. annealing at 60 °C, 45 s. extension at 72 °C, and 5 min. final extension at 72 °C. Primers Exon6F and Exon6R amplify 510 bp long wild-type allele while primers NEO-F and NEO-R amplify 340 bp long knockout allele. Genotype of each mouse used in this study was determined before experiments and confirmed at the end of each experiment.

### Home cage monitoring

We used the Home Cage Scan system from CleverSys^14–18^ consisting of 4 cameras simultaneously monitoring 4 mice individually housed in separate cages on a 12:12 light: dark cycle. The cage environment was lit by white lights from 6 AM to 6 PM and infrared lights from 6 PM to 6 AM. The computer software allows the researcher to calibrate the cage, providing software with information about the cage environment such as the top/bottom of cage, height of bedding, placement of drinking spout, and food bin areas. The software then uses this information to recognize the animal and behavior in durations greater than 6 frames per second.

Eight 6-week-old *Nags*^−/−^ and eight aged-matched wild-type controls were used for this study. Mice were housed with minimal bedding to reduce mounding, which can obscure the mouse during recording. Mice were acclimated to their cages and the environment for 48 h prior to the study and were recorded for 23 h beginning at either 10:00 AM or 4 PM. Mice remained on NCG + Cit supplemented water for the first 23 h. After the first 23 h, the NCG + Cit supplemented water was replaced with drug-free water before the next 23 h of recording. Recording of behavior lasted for 23 h in order to give the researcher time to change the water and recalibrate the cages so that day 2 recording could also start at either 10:00 AM or 4 PM. Random bouts of movies were inspected after the experiment to determine the accuracy of the behaviors called by the recognition software. Incorrect behaviors were manually changed by the researcher. Results are presented as the percent of animals displaying a behavior.

The software provides the researchers with more than 30 different behaviors. We chose to group some of these behaviors into seven categories for data analysis. The software recognizes and distinguishes the behaviors rear up, come down from partially reared, come down to partially reared, rear up from partially reared, rear up to partially reared, remain reared up, and remain partially reared, which we grouped as “Rear up”. “Hanging” regroups the behaviors hang cuddled, hang vertical from hang cuddled, remain hang vertical, and remain hang cuddled as defined by the software. “Eat” regroups the software distinguished behaviors eat and chew. “Walk” regroups the software distinguished behaviors walk left, walk right, and walk slowly (Table S1).

### Measurements of mouse activity with voluntary wheels

Eight mice per experimental group were kept individually in cages equipped with a running wheel with the magnet (Minimitter Company, Inc. Surriver or Vers 4.1). Running wheel system was monitored by online computer using Vital View data acquisition system (StarrLife, Inc). The number of turns of each wheel was recorded in 1 min. bins. The running distance and velocity were calculated based on a 0.36 m wheel circumference. After transfer from their home cages to cages with voluntary wheels, mice were allowed 48 h to acclimate to the new environment. Following acclimation period, the activity of the mice supplemented with NCG + Cit in the drinking water was recorded for 48 h; NCG was removed from the drinking water on the third day at 12 PM, and activity was recorded for additional 48 h. Mice that became inactive due to hyperammonemia were euthanized, and their plasma and livers were collected and stored at − 80 °C for molecular and biochemical analyses. Physical activity was measured as running time, distance, and velocity.

### Plasmid construction and AAV vector preparation

The 1593 bp mNAGS coding sequence identical to GenBank ID AF462069.1 was synthesized by DNA2.0, Inc. The AAV2/8.TBG.mNAGS plasmid was generated by inserting mNAGS coding sequence into AAV2/8.TBG plasmid^32^ using restriction endonucleases *Not*I and *Bgl*II. Correct sequence between AAV inverted repeats of the AAV2/8.TBG.mNAGS construct was verified by DNA sequencing. For the AAV2/8.NAGS.mNAGS construct TBG promoter was replaced with mouse *Nags* promoter^47^. Mouse genomic DNA was extracted following the standard protocol using the DNeasy Blood and Tissue Kit, Qiagen (Valencia, CA). The 684 bp fragment harboring mouse *Nags* promoter was amplified using primers 5′-CAG ATCCGGCGCGCCCTCTCTATAATATGTAGCCCC-3′ and reverse primer 5′-GACCAACTTCTGCAGGACGACAACCAAACCCACTCG-3′, and the following PCR conditions: 5 min initial denaturation at 95 °C followed by 30 cycles of 30 s denaturation at 95 °C, 30 s annealing at 62.5 °C, 1 min extension at 72 °C and 7 min. final extension at 72 °C. The TBG promoter of the AAV2/8.TBG.mNAGS plasmid was replaced with the amplification product using In-Fusion Cloning Kit (Clontech) according to manufacturer’s instructions. Correct sequence between AAV inverted repeats of the AAV2/8.NAGS.mNAGS construct was verified by DNA sequencing. The E354A mutation was introduced using 5′-GCA CGC TGC TCA CGG CAC TCT TTA GTA ACA AGG GC-3′ mutagenic primer and the QuikChange Lightning Site-directed mutagenesis kit (Agilent) according to manufacturer’s instruction. Correct sequence between AAV inverted repeats of the resulting AAV2/8.NAGS.mNAGS-E354A construct was verified by DNA sequencing. QIAGEN Plasmid Giga Kit was used to purify approx. 2.4 µg/µl of AAV2/8.TBG.mNAGS, AAV2/8.NAGS.mNAGS and AAV2/8NAGS.mNAGS-E354A plasmids. AAV particles containing each of these three plasmids were prepared by the Penn Vector Core, Gene Therapy Program, University of Pennsylvania School of Medicine, USA.

### Delivery of mNAGS into Nags^−/−^ mice

AAV viral particles were stored as suspension in sterile phosphate buffered saline (PBS) with 5% glycerol at − 80 °C. The viral particle suspension was thawed, diluted to concentrations indicated in the text, and delivered via tail vein injections of 100 µl particle suspension. After the injection experimental mice were kept in the home cages for five days prior to voluntary wheel experiments. In mRNA and protein persistence experiments, mice were kept in home cages after injection of the AAV viral particles. Mice were supplemented with NCG + Cit during this time.

### Plasma ammonia and amino acid analysis

Blood was collected from euthanized mice by cardiac puncture. Blood was immediately centrifuged at 7500×*g* for 5 min at 4 °C, and the plasma was stored at − 80 °C until further analysis. The Ammonia Assay Kit (Abcam, Cambridge, MA) was used to determine plasma ammonia according to the manufacturer’s protocol.

Plasma amino acid concentrations were measured using ion-exchange chromatography on a High-Speed Amino Acid Analyzer L-8800/L-8800A (Hitachi). Plasma proteins were precipitated with an equal volume of 7% sulfosalicylic acid and centrifuged for 10 min at 13,000 rpm. 5 μl of 2.5 N LiOH and 10 μl stock internal standard (S-2-aminoethyl-L-cysteine hydrochloride) were then added to 100 μl of plasma supernatant before loading samples into the analyzer. Amino acids were quantified using L-8800 ASM software package.

### RNA extraction and real time PCR

RNA extraction was performed as per the manufacturer’s instruction using RNeasy Mini Kit (Qiagen, USA). A total of 2 ug of RNA was transcribed into cDNA using the High-Capacity cDNA reverse Transcription kit (Applied Biosystems, USA) according to the manufacturer’s instructions. For the real time PCR analysis, 1 µl of mNAGS gene probe Mm00467530_m1 (TaqMan), 10 µl of TaqMan Gene Expression Master Mix and 4 µl of cDNA were combined in 20 µl reaction and subjected to PCR using AB 7900 Real-Time PCR System. Abundance of mNAGS mRNA was determined using ∆∆C_t_ method^48^; 18S rRNA gene was used for normalization.

### Immunoblotting

Frozen liver samples were pulverized with liquid nitrogen and the cells were lysed using lysis buffer (50 mM Tris pH 7.5, 1 mM EDTA, 1% NP40, 250 mM Sucrose, 0.1% SDS and Protease Inhibitor Cocktail tablets, Roche). Protein quantification was performed using the Quick Start™ Bradford Protein Assay (Bio-Rad) according to manufacturer’s instructions. 50 µg of proteins from the liver lysate were resolved using 4–20% gradient polyacrylamide gel (Bio-Rad, USA) and transferred to nitrocellulose membrane using Trans-Blot Turbo Transfer System (Bio-Rad, USA). Membranes were then cut in two pieces, one having proteins with molecular weights above 75 kDa and the second one having proteins with molecular weights below 75 kDa followed by blocking of both with the Starting Block (TBS) Blocking buffer (Thermo Scientific, Rockford, IL) with 0.5% Surfact-Amps 20 (Thermo Scientific, Rockford, IL) overnight at 4 °C. Blocked membranes containing proteins with molecular weights above 75 kDa were probed with the mouse anti-vinculin (1:2000 dilution) monoclonal primary antibody (Sigma-Aldrich) while proteins with molecular weight below 75 kDa were probed with the rabbit anti-mNAGS antibody (1:3000 dilution) for 1 h at room temp. Primary anti-mNAGS antibody was detected with the peroxidase conjugated goat anti-rabbit (1:10,000 dilution) secondary antibody (Thermo Scientific, Rockford, IL), and was visualized using Super Signal West Pico Chemiluminescent Substrate (Thermo Scientific, Rockford, IL). Primary anti-vinculin antibody was detected with the peroxidase conjugated anti-mouse IgG (1:4000 dilution) secondary antibody (Cell Signaling Technology) and visualized using Super Signal West Pico Chemiluminescent Substrate. The band intensities were quantified using ChemiDoc™ Touch Imaging System instrument with Image Lab software, version 5.2.1 (Bio-Rad, USA).

### Plasmid construction, recombinant protein overexpression and purification

Plasmids pET15bhNAGS-M and pET15bmNAGS-M^37^ were used for overexpression of mNAGS and hNAGS in *E. coli*. Mutagenic primers 5′-GCA CGC TGC TCA CTG ACC TCT TTA GCA ACA AGG-3′ and 5′-GCA CGC TGC TCA CGG ACC TCT TTA GTA ACA AGG-3′ were used to generate constructs pETbhNAGS-E360D and pET15bmNAGS-E354D using QuikChange Lightning Site-directed mutagenesis kit (Agilent) according to manufacturer’s instruction. Recombinant NAGS proteins were purified using nickel affinity chromatography as described previously^18,49^. Quality of purified proteins was assessed using SDS-PAGE.

### Enzyme activity measurements and Thermofluor^®^ analysis

Specific activities of purified enzymes were measured as described previously^50^. Thermal shift assays were performed in a 96 well plate format using a QuantStudio 7Flex Real-Time PCR System (Applied Biosystems). Protein unfolding was monitored by measuring the change in fluorescence intensity of SYPRO Orange (Invitrogen) while ramping temperature from 4 to 99 °C. Wells contained 1 μg of enzyme in 50 mM potassium phosphate pH 7.5, 300 mM KCl, 20% glycerol, 250 mM imidazole, 10 mM β-mercaptoethanol (BME), 0.006% Triton X-100, 1% acetone, 50× SYPRO Orange and 10 mM of either L- or D-arginine.

### Statistical analysis

The Kaplan–Meier estimator was used to analyze the time to cessation of activity on the voluntary wheel. Two-way ANOVA was used to analyze differences in activity on the voluntary wheel. The Mann–Whiney test was used to analyze differences in the length of running sessions, running distance and velocity for during each session. All other data were analyzed using Student’s t-test.

## Supplementary Information

Supplementary Information
