# Supplementary material for: Gene delivery corrects N-acetylglutamate synthase deficiency and enables insights in the physiological impact of L-arginine activation of N-acetylglutamate synthase
Source: Sci Rep. 2021 Feb 11;11:3580. doi: 10.1038/s41598-021-82994-8 (PMC7878489; doi:10.1038/s41598-021-82994-8)
Supplement: Supplementary file 1 — Supplementary Information [file 41598_2021_82994_MOESM1_ESM.pdf]

Gene Delivery Corrects N-Acetylglutamate Synthase Deficiency and Enables Insights in the Physiological Impact of L-arginine Activation of N-Acetylglutamate Synthase

Sonaimuthu P[1], Senkevitch E[1], Haskins N[1], Uapinyoying P[1,2]; McNutt M[3], Morizono H[1], Tuchman M[1], Caldovic L[1]*

[1]Center for Genetic Medicine Research, Children's National Medical Center, Washington DC, [2]National Institute of Neurological Disorders and Stroke, National Institutes of Health, Bethesda, MD; [3]Children's Medical Center, UT Southwestern Medical Center, Dallas, TX

*Corresponding Author: Ljubica Caldovic, PhD
Children's Research Institute
Children's National Medical Center
111 Michigan Ave NW
Washington DC, 20010
Tel: (202) 476-5819
Fax: (202) 476-6014
Email: LCaldovic@childrensnational.org

Keywords: urea cycle, ureagenesis, N-acetylglutamate synthase, N-acetylglutamate, arginine, regulation of ureagenesis, AAV-based gene transfer

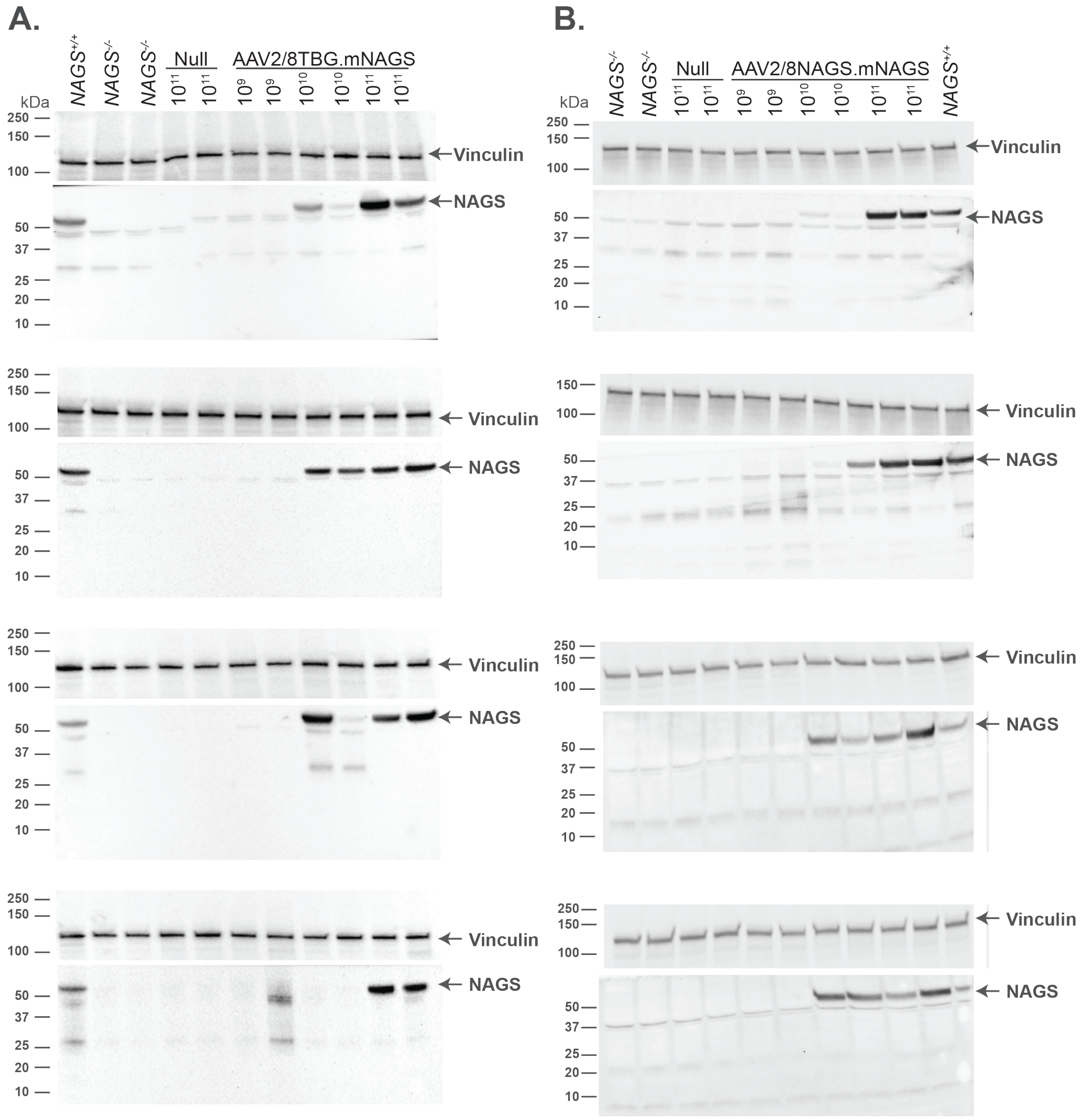

Figure S1. Full length immunoblots of the NAGS protein in the livers of mice injected with increasing doses of either AAV2/8TBG.mNAGS (**A**) or AAV2/8NAGS.mNAGS (**B**) vector. AAV2/8TBGnull (Null) vector was injected as a control. Vinculin was used as a loading control.

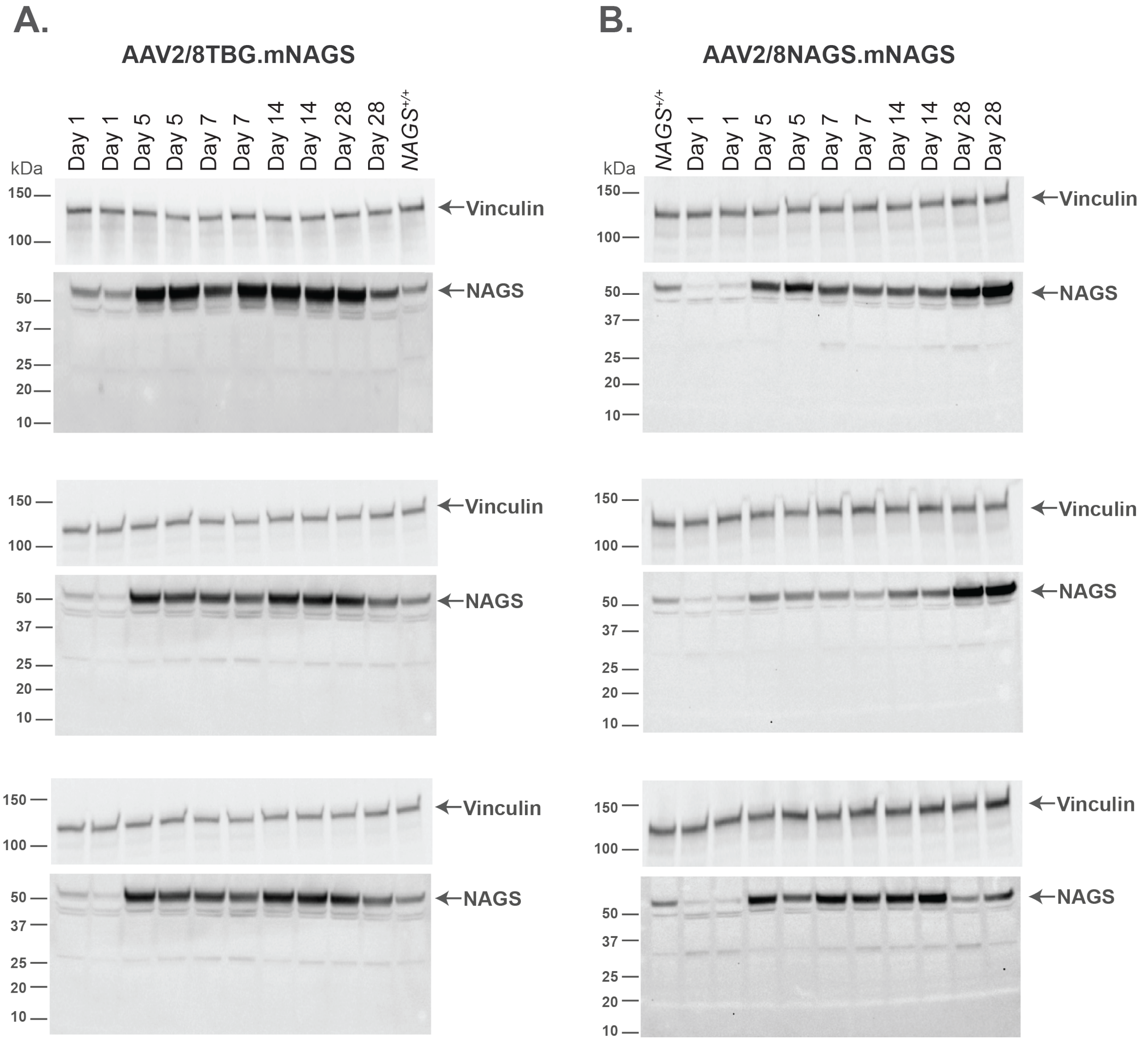

Figure S2. Full-length immunoblots of the NAGS protein in the livers of mice after single injection with 10^{11} particles of either AAV2/8TBG.mNAGS (**A**) or AAV2/8NAGS.mNAGS (**B**) vector. Vinculin was used as a loading control.

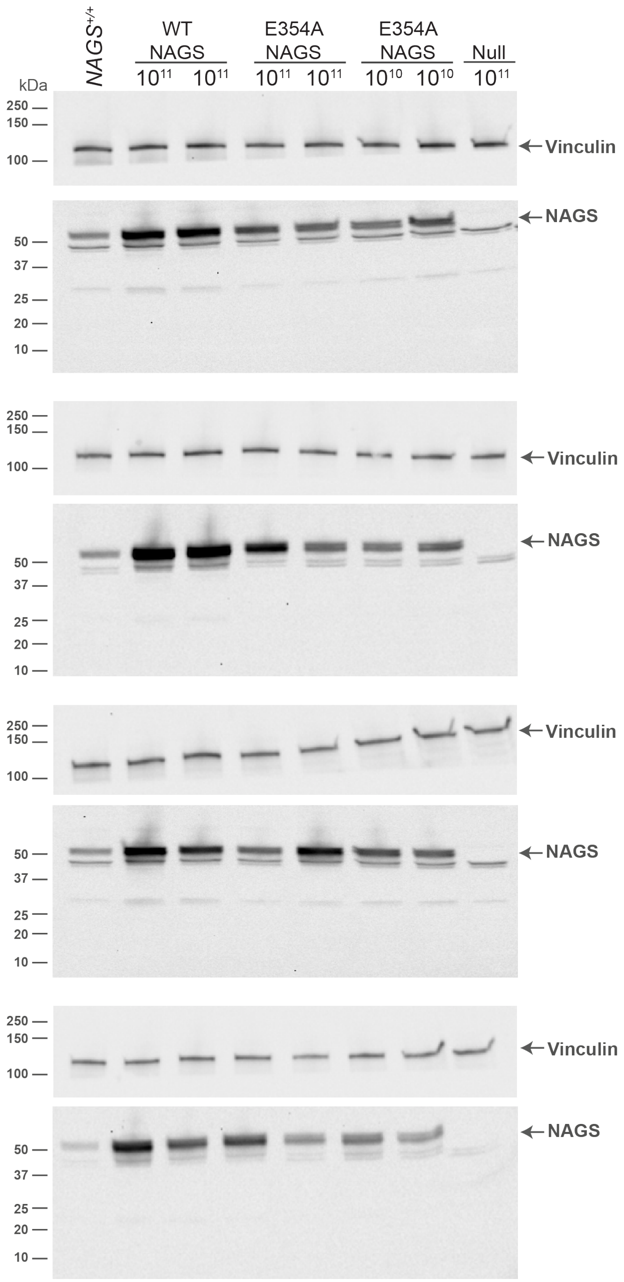

Figure S3. Full-length immunoblots of the NAGS protein in the livers of mice injected with 10^{11} viral particles of the AAV2/8NAGS.mNAGS vector (WT NAGS lanes), with either 10^{11} or 10^{10} viral particles of the AAV2/8NAGS.E354A-mNAGS vector (E354A NAGS lanes), or with 10^{11} viral particles of the AAV2/8TBGnull vector (Null). Vinculin was used as a loading control.

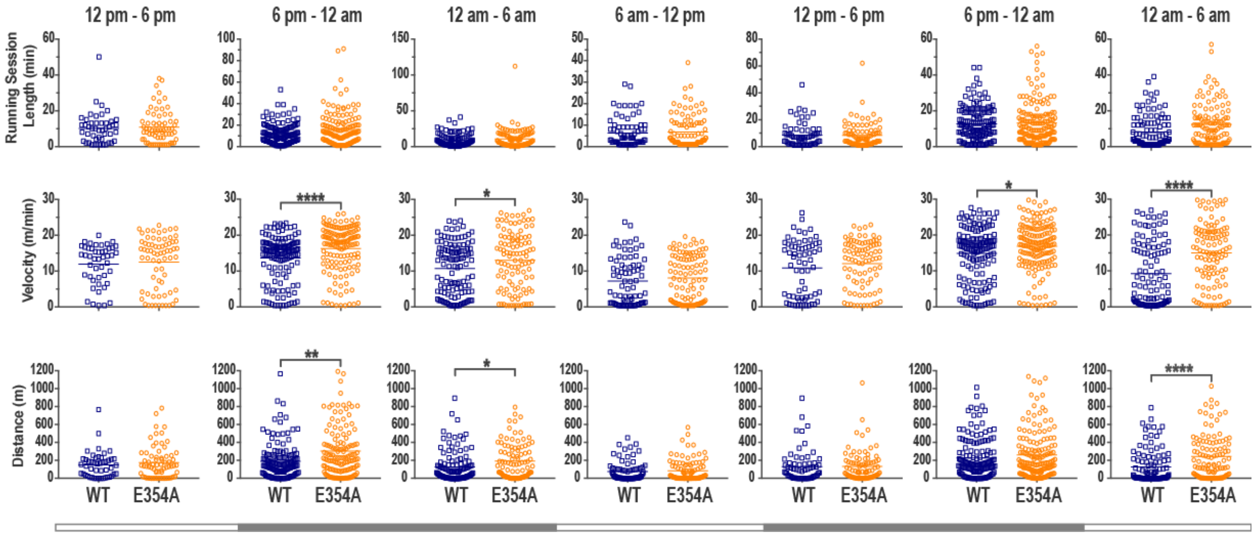

Figure S4. Length of running sessions, velocity and distance during each running session in 6 hr. epochs after withdrawal of NCG supplementation. The line below graphs indicate light (open) and darkness (gray) periods. Orange – AAV2/8NAGS.mNAGS-E354A. Blue – AAV2/8NAGS.mNAGS. Shaded bars indicate nighttime when mice are more active. ∗ – p<0.01, ∗∗ – p<0.001, ∗∗∗∗ – p<0.0001.

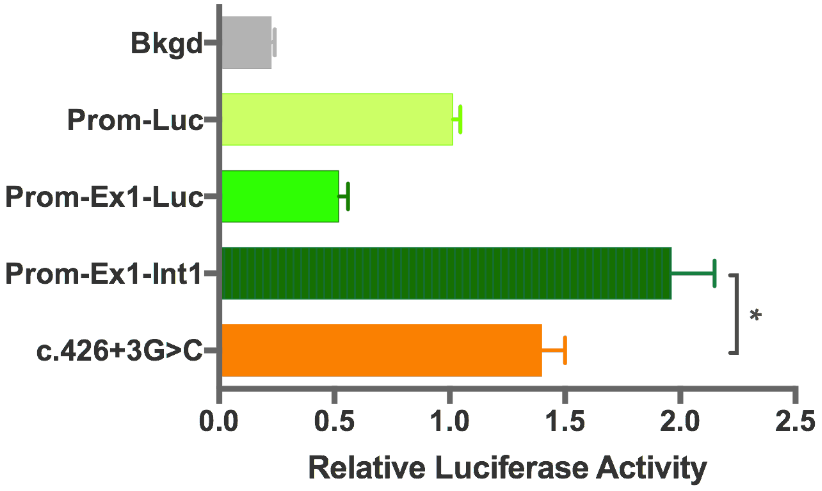

Figure S5. Effect of the *NAGS*:c.426+3G>C sequence variant on gene expression in HuH-7 cells. Bkgd – luciferase activity in mock transfected cells. Prom-Luc – luciferase activity in HuH-7 cells transfected with construct harboring *NAGS* promoter upstream of the luciferase reporter gene. Prom-Ex1-Luc – activity of luciferase protein fused to protein sequence of the *NAGS* exon 1. Prom-Ex1-Int1 – luciferase activity in HuH-7 cells transfected with construct harboring *NAGS* exon 1 and intron 1 fused to luciferase reporter gene. C.426+3G>C – luciferase activity in HuH-7 cells transfected with the mutated Prom-Ex1-Int1 construct. Asterisk indicates $p<0.05$.

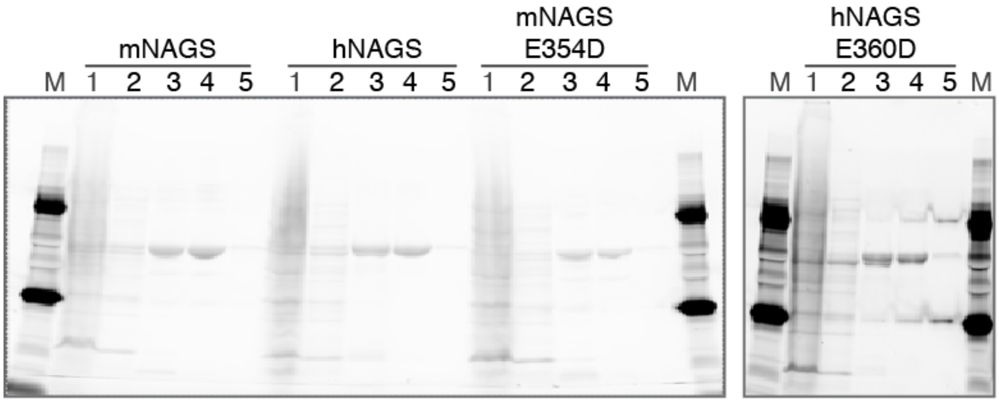

Figure S6. SDS-PAGE gels of samples from various stages of the purification of either wild type or mutant human and mouse NAGS (hNAGS and mNAGS, respectively). Lane 1 – cell lysate, lane 2 – flow-through, lane 3 – elution with 125 mM imidazole, lane 4 – elution with 250 mM imidazole, lane 5 – elution with 500 mM imidazole, M – molecular size markers.

Table S1. Grouping of mouse behaviors prior to their quantitative analysis.

**Mouse behaviors defined by Home Cage Scan program:**	**Grouped by researcher for analysis as:**
Rear Up	Rear Up
Come down from partially reared	
Come down to partially reared	
Rear up to partially reared	
Remain reared up	
Remain partially reared	
Hang cuddled	Hanging
Hang vertical from hang cuddled	
Remain hang vertical	
Remain hang cuddled	
Eat	Eating
Chew	
Drink	Drinking
Walk left	Walking
Walk right	
Walk slowly	
Groom	Grooming

Supplementary Materials and Methods.

Reporter gene plasmid construction

Construction of the plasmid 4.10Prom, which has been renamed to Prom-Luc in this study, was described previously[1]. To generate plasmid Prom-Ex1-Luc sequence of first exon of the human *NAGS* gene was amplified using 5'-AAG CTT ATG GCG ACG GCG CTG ATG GCT-3' and 5'-CCA TGG CTC GAT GAC GGC GAA GGG CTT-3' primers and Platinum Taq PCRx DNA polymerase (ThermoFisher) according to the manufacturer's instructions. The amplicon was subcloned into Prom-Luc plasmid using *Hind*III and *Nco*I restriction endonucleases. This created a 2 bp insertion between DNA sequences of the NAGS exon 1 and luciferase genes. The 2 bp insertion was removed using QuickChange Site-Directed Mutagenesis kit (Agilent Technologies) and mutagenic primer 5'-CCT TCG CCG TCA TCG AGA TGG AAG ATG CCA AAA A-3' and 5'-TTT TTTG GCA TCT TCC ATC TCG ATG ACG GCG AAG G-3' according to manufacturer's instructions. To generate plasmid Prom-Ex1-Int1-Luc, a minigene containing portion of the NAGS exon 1 downstream of the *Sbf*I restriction site, entire first intron of the NAGS gene, first 6 bp of the NAGS exon 2 and a portion of the luciferase gene upstream of the *Age*I site was synthesized by GeneScript (www.genescript.com) and subcloned into Prom-Ex1-Luc plasmid using *Sbf*I and *Age*I restriction enzymes. Mutagenic primers 5'-GCT CCG CTG ACC TCG ATG ACG GCG-3' and 5'-CGC CGT CAT CGA GGT CAG CGG AGC-3' were used to introduce the c.426+3G>C sequence variant into Prom-Ex1-Int1-Luc plasmid. Correct sequence pf all plasmids was verified by DNA sequencing prior to transfection into cultured cells.

Cell culture and transfection

Human hepatocarcinoma HuH-7 cells (Charles Rice laboratory, Rockefeller University) were cultured as described previously[2]. Cells were plated at a density of 1.5×10^5 cells/well on 24-well culture plates 24 hours prior to transfection. The cells (90–95% confluent) were then transfected using Lipofectamine 3000 reagent (Life Technologies) and cultured in transfection media containing medium and serum only. A total of 0.5 μg of DNA was transfected with 0.495 μg of vector expressing luc2 and 0.005 μg of pGL4.74 vector containing *Renilla reniformis* luciferase (hRluc) as an internal control (Promega).

Reporter gene assays

24 hours following transfection, cells were assayed for both firefly and Renilla luciferase activity using Dual-Luciferase Reporter Assay System (Promega) and Berthold Centro 960 luminometer (Berthold) according to the manufacturer's protocol. The firefly luciferase signals were normalized to Renilla luciferase activity to correct for differences in transfection efficiency. Expression levels of each construct were then normalized to luciferase activity of the Prom-Luc plasmid. All results are an average of three

independent experiments that were each carried out in triplicate. Values were expressed as mean ± SEM and analyzed using Student's t-test.

1 Heibel, S. K. *et al.* Transcriptional regulation of N-acetylglutamate synthase. *PLoS One* **7**, e29527, doi:10.1371/journal.pone.0029527 (2012).

2 Jang, Y. J. *et al.* Disease-causing mutations in the promoter and enhancer of the ornithine transcarbamylase gene. *Hum Mutat* **39**, 527-536, doi:10.1002/humu.23394 (2018).
